# Getting stuck in a rut as an emergent feature of a dynamic decision-making system

**DOI:** 10.1098/rsos.231550

**Published:** 2024-04-03

**Authors:** Matthew Warburton, Jack Brookes, Mohamed Hasan, Matteo Leonetti, Mehmet Dogar, He Wang, Anthony G. Cohn, Faisal Mushtaq, Mark Mon-Williams

**Affiliations:** ^1^ School of Psychology, University of Leeds, Leeds, UK; ^2^ School of Computing, University of Leeds, Leeds, UK; ^3^ Centre for Immersive Technologies, University of Leeds, Leeds, UK; ^4^ Max Planck UCL Centre for Computational Psychiatry and Ageing Research, University College London, London, UK; ^5^ Department of Informatics, King’s College London, London, UK; ^6^ Centre for Applied Education Research, Wolfson Centre for Applied Health Research, Bradford Teaching Hospitals NHS Foundation Trust, Bradford, West Yorkshire, UK; ^7^ National Centre for Optics, Vision and Eye Care, University of South-Eastern Norway, Kongsberg 3616, Norway

**Keywords:** decision-making, hysteresis, choice bias

## Abstract

Human sensorimotor decision making has a tendency to get ‘stuck in a rut’, being biased towards selecting a previously implemented action structure (hysteresis). Existing explanations propose this is the consequence of an agent efficiently modifying an existing plan, rather than creating a new plan from scratch. Instead, we propose that hysteresis is an emergent property of a system learning from the consequences of its actions. To examine this, 152 participants moved a cursor to a target on a tablet device while avoiding an obstacle. Hysteresis was observed when the obstacle moved sequentially across the screen between trials, whereby the participant continued moving around the same side of the obstacle despite it now requiring a larger movement than the alternative. Two further experiments (*n* = 20) showed an attenuation when time and resource constraints were eased. We created a simple computational model capturing probabilistic estimate updating that showed the same patterns of results. This provides, to our knowledge, the first computational demonstration of how sensorimotor decision making can get ‘stuck in a rut’ through the updating of the probability estimates associated with actions.

## 1. Introduction

Humans are creatures of habit and often repeat behaviours—despite the selected action having a higher cost than an available alternative. This propensity can be seen when we continue to use the road well-travelled when moving between two buildings even after construction work has created a shorter route. The phenomenon is particularly remarkable because adult humans are generally so adept at selecting optimal movement patterns [1]. Indeed, the ability of humans to rapidly and efficiently execute actions far exceeds the capabilities of even the most sophisticated robotic systems [2]. The incredible repertoire of skilled behaviour in humans reflects the presence of learning processes that have been trained over the countless occasions when adults have interacted with the external world. These myriad interactions allow the human nervous system to accurately estimate the costs associated with various behaviours and thereby select an optimal (or close to optimal) action when presented with a goal-directed task. Yet humans will select different options on different occasions as a function of whether the choice is made de novo or following a previous successful action—despite the choices having the same relative costs on both occasions.

The tendency to show a bias towards a previously selected action plan can be described as ‘hysteresis’ (or the sequential effect) and is well-studied. Hysteresis effects have been found both for binary decisions, like grasp selection [3–9] and hand selection [10–12], as well as in continuous domains, like hand path priming experiments [13–15] and grasp height [16] and angle [17]. The predominant explanation for hysteresis is teleological in nature: it is proposed that modifying a previously used action is more cognitively efficient than planning from scratch, so hysteresis exists to increase planning efficiency [11,18–20], as indexed by reduced reaction times (RTs) when using the same action as previously [21], and a model shows how this theory can operate in practice for continuous behaviours [20]. While it is possible that hysteresis as observed in discrete and continuous choices operates through different mechanisms, we believe another simple principle can explain the phenomenon in both domains.

We propose that the process of ‘getting stuck in a rut’ is an emergent property of a decision-making system that dynamically learns from the consequences of its actions. In order to deal with unpredicted changes in the world (and adapt to novel environmental states), an efficient system must frequently update its estimates of the success probabilities associated with a given action through the use of feedback about the outcomes of their actions. We suggest that this principle—the updating of success probabilities—will naturally result in a system that shows hysteresis. Indeed, previous work has shown that hand choice is biased by recent successes when the probability of success is surreptitiously manipulated [22], and endpoint variability of reaches are lower when movements are made to the same target consecutively compared to movements to a new target [23].

It is common practice in computer science to model the environment as a partially observable Markov decision process (POMDP; [24]) when designing agents that need to act under uncertainty, whereby the agent does not directly observe the environment’s state but receives a state-dependent observation following an action executed by the agent, and must use the actions and observations to learn the optimal control policy for given state beliefs. POMDPs reflect well the challenges faced by the human nervous system which must infer the hidden states of the environment from the sensory inputs that follow an action [25]. The important point from the perspective of this manuscript is that the external world is not static and this means that a human agent must frequently update its internal representation in order to act optimally in a noisy and changing world. The ability to make accurate predictions allows a human to generate an action that will produce a desired change in the sensory input, and we hypothesized that the updating of predictions following sensory observations would produce hysteresis as an emergent property of the learning system.

In order to explore hysteresis, we designed a sensorimotor decision-making task that would allow us to vary critical task parameters and observe behaviour on a trial-by-trial basis to understand the frequency of updating. Participants needed to move around an obstacle (left or right) to hit a target (where the reward was the same for each choice). We manipulated the obstacle position on each trial across blocks such that it either moved systematically across the screen or was randomly positioned across trials. Based on previous work, we predicted that participants would show a strong bias towards repeating previously selected actions in the sequential condition, even when the obstacle position indicated an alternative route would be preferable, whereas no global hysteresis effect would emerge when the obstacle was positioned randomly between trials. To understand whether the observed data could be explained by a POMDP-inspired model, we created a model that included a trial-by-trial update of the success probabilities associated with one action versus another. The goal of the model was to simulate how an agent would respond to a choice between two options that both allow a given goal to be achieved but with different costs.

Converging evidence suggests that the decision-making process is governed by neural circuits that accumulate noisy evidence for possible options over time, with a decision triggered when sufficient evidence is accumulated to cross an action threshold [26–28]. Under such a process, actions will be selected more rapidly (i.e. RTs will decrease) when there is a bias towards one action versus another, either through biasing the starting amount of evidence or the rate of evidence accumulation. This suggests that RTs will be faster in the presence of hysteresis, consistent with previous observations [21], but that this is a useful by-product of a dynamic decision-making system rather than the planned product of a system designed to produce hysteresis.

On the assumption that evidence accumulation processes are a core component of sensorimotor decision making, we anticipated finding RT differences as a function of the magnitude of hysteresis associated with a given task. We further hypothesized that relaxing the temporal constraints of the task would attenuate the size of the hysteresis effect (as the available time can be used to more fully evaluate the costs of either action, such that any biases are less impactful). In experiment 2, we directly manipulated the temporal constraints of the task by creating a ‘waiting period’ before which an action could be executed. In experiment 3, we indirectly manipulated the temporal constraints by decreasing the ‘higher order’ cognitive demands of the task. We reasoned that decreasing the cognitive constraints would allow the task goal to be identified more rapidly and thereby create a longer period in which the respective costs of the alternative actions could be computed.

## 2. Methods

### 2.1. Participants

In experiment 1, 152 adults (41 males, 100 females; mean age 22.51 years, range 18–39 years; 139 self-reported right-handed; 11 participants did not report age or gender) were recruited as part of a larger motor control project. Participants for experiment 2 (*n* = 20, one male, 19 females; mean age 19.09, range 18–20 years; 20 self-reported right-handed) and experiment 3 (*n* = 20, one male, 19 females; mean age 18.86 years, range 18–20 years; 18 self-reported right-handed) were recruited through word of mouth from the University of Leeds undergraduate population. All had normal or corrected-to-normal vision and provided informed consent to participate. Participation in these studies was incentivized through remuneration of £2 on completion of the experiment. Ethical approval was obtained from the University of Leeds ethics committee.

### 2.2. Procedure

**Figure 1.**
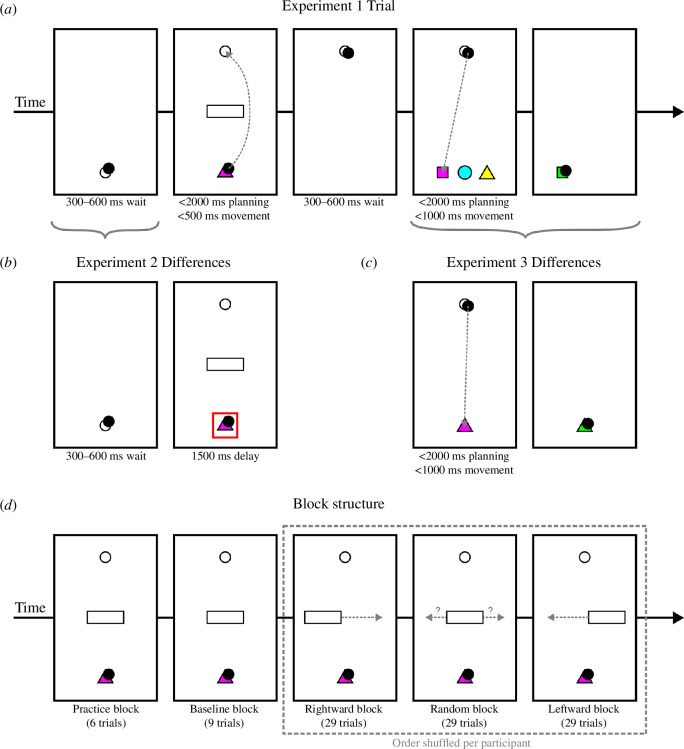
Trial and block structure of the experiments. (*a*) A complete trial for experiment 1. In step (i), participants moved to a start point and waited 300–600 ms until it changed to a colour and a shape indicating the target shape colour. Simultaneously, an obstacle and a checkpoint appeared. Participants were allowed 2000 ms planning time at the start point before moving around the obstacle to the checkpoint (<500 ms) and waited 300–600 ms until three targets appeared. Participants were allowed up to 2000 ms in the checkpoint before moving to the target that matched the colour shown at the start point (<1000 ms). (*b*) For experiment 2, step (i) of experiment 1 was replaced by two steps. Participants moved to a start point and were immediately shown a red box around the start point, indicating they could not leave. After a random 300–600 ms delay, the stimuli were revealed but the red box remained on screen for a further 1500 ms. (*c*) For experiment 3, steps (iv) and (v) of experiment 1 were changed so only one target was revealed, of the same colour and shape as the start point. (*d*) The block structure of the experiments. Participants completed a practice and baseline block, where the obstacle was always central to the screen, before completing a shuffled order of the rightwards block (obstacle moves from the left to the right between trials), leftwards block (the obstacle moves from the right to the left between trials) and random block (obstacle moves randomly between trials).

Participants sat at a desk with a touchscreen computer tablet (Lenovo ThinkPad Helix 2, 1920 × 1080 pixels, 11.6″ screen, 60 Hz refresh rate) placed directly in front of them and interacted with the screen using a stylus (sampled from the screen digitizer at a rate of 100 Hz) in their chosen hand. Participants were shown a pictorial instruction sheet prior to starting the experiment that explained how to complete a single trial. Prior to starting the experiment, participants were instructed that each trial should be completed as quickly and accurately as possible. The core trial structure was the same across all three experiments and key differences for each study are detailed below and presented in figure 1.

#### 2.2.1. Experiment 1: biases in sensorimotor decision making

In experiment 1, we introduced a novel sensorimotor decision task in which participants were asked to select one of two possible routes around an obstacle to reach a target using a stylus on a tablet display. The experimental task was developed using Unity (v. 2018.1) and the Unity Experiment Framework [29]. During each trial, participants had to stay within a 200 mm high by 106 mm wide workspace, displayed as a rectangle on the screen. Participants began each trial by placing the stylus on the screen and moving the cursor (5 mm diameter circle) to a start point (10 mm diameter circle) at the bottom of the screen. After a uniformly sampled random delay between 300 and 600 ms (to avoid anticipation of trial start), the start point changed to a colour and shape combination, randomly selected from a list of three of each (cyan, magenta and yellow; circle, square and triangle); a checkpoint (10 mm diameter circle) appeared in the top of the screen; and an obstacle (30 mm wide × 10 mm high rectangle) appeared equidistant between the start point and checkpoint. The distance between the start point and checkpoint was 140 mm. Participants were instructed to remember the colour they were shown and move as quickly and accurately after the stimulus display to the checkpoint. Participants were allowed up to 2 s ‘preparation time’ at the start point. Upon leaving the start point the coloured shape disappeared, and the participant had up to 500 ms to reach the checkpoint. Upon entering the checkpoint, the obstacle disappeared. The participant then had to wait at the checkpoint. After a uniformly sampled random delay between 300 and 600 ms, three targets (with randomly assigned colour and shape combinations) appear at the bottom of the screen. The vertical distance between the checkpoint and targets was 150 mm, with 30 mm spacing between targets horizontally. Participants were instructed to move as quickly and accurately to the target of the same colour that was shown at the start point. Participants were allowed up to 2 s in the checkpoint, and had up to 1 s to reach the target upon leaving the checkpoint.

A trial was successfully completed when the participant moved to the correct target. There were several ways to fail a trial: hitting the obstacle or task boundaries; leaving the checkpoint before the targets were revealed; moving to the wrong coloured target; or taking too long during any timed period. If a failure was triggered the trial was immediately terminated. Once a trial finished, visual feedback was presented for 1 s to indicate the outcome of a trial, where the target turning green indicated a successful trial, and an object turning red indicated a failed trial. For failed trials, the object that turned red indicated the type of failure (e.g. if the participant hit the obstacle, it would turn red). Across all trials, a running score was shown in the top left of the screen, which increased by 1 after each successful trial. After visual feedback had been presented for 1 s, the start point was shown on the screen, and participants were able to begin a new trial.

The experiment comprised a total of 106 trials and took approximately 10 min to complete. This included four example trials, six practice trials, nine baseline trials and 87 experimental trials made up of three blocks of 29 trials, shown in figure 1*d*. During the example trials, the instructor showed the participant a set of four standard trials including two successful trials and two failed trials. Participants were provided six practice trials to make sure they understood the task mechanics, which included text feedback after every trial to indicate the outcome, in addition to the regular visual feedback. A baseline block followed that aimed to make participants move as quickly as they could (while maintaining accuracy) by giving text feedback telling them they needed to move more quickly if their movement time between the start point and checkpoint was slower than their previous fastest time. During each of these three blocks, the obstacle was located horizontally central to the screen, and after the completion of each block, text was displayed for 10 s on the screen to indicate the participant was starting a new phase of the experiment.

The experimental trials were then organized into three blocks, presented to the participant as one uninterrupted block. The three conditions were where the obstacle’s horizontal position moved sequentially between trials from the left of the screen to the right (rightwards), from the right of the screen to the left (leftwards), and where the obstacle’s positions were randomly shuffled (random). Twenty-nine obstacle positions were used with extreme positions of −34.2 mm and 34.2 mm, with equally spaced jumps between each position, and each obstacle position was tested once per block. The order of the three conditions was randomly allocated per participant (we found no consistent effect of condition order on hysteresis magnitude, though participants in one ordering [*rightwards–leftwards–random*] showed a slightly lower magnitude relative to the other five orders).

#### 2.2.2. Experiment 2: decreasing temporal constraints

Experiment 2 was conducted to explore whether easing the temporal constraints placed on the decision-making system could attenuate hysteresis. Upon entering the start point a red box appeared surrounding the start point and the participant’s cursor. While the red box was visible the participant was not allowed to leave the start point or the trial would terminate in failure. In common with experiment 1, there was a randomly sampled delay of between 300 and 600 ms before the stimuli were presented. However, in experiment 2, the red box remained on the screen after the stimulus display. After 1.5 s the red box disappeared, and the participant completed the rest of the trial as described in experiment 1. Differences between these experiments are illustrated in figure 1*b*.

#### 2.2.3. Experiment 3: reducing task cognitive demands

Experiment 3 was conducted to explore whether reducing the cognitive demands associated with the task could attenuate hysteresis. Participants were presented with only one target after waiting at the checkpoint, with the colour shown at the start of the trial always matching that of the target. The trial was otherwise identical to experiment 1 (differences illustrated in figure 1*c*).

### 2.3. Data analysis

Stylus position data, sampled at 100 Hz, were filtered using a dual-pass Butterworth second-order filter with a cut-off frequency of 10 Hz. To detect movement onset, the time when movement speed rose above 50 mm s^-1^ closest to the Unity’s timestamp of the participant leaving the start point was classed as movement start. For experiments 1 and 3, RT was calculated as the difference in time between the obstacle being shown and movement start, whereas for experiment 2, it was the difference between the red box disappearing and movement starting. RT data were preprocessed by removing trials where no RT was present (117 trials, 0.7%), where RTs were lower than 100 ms (36 trials, 0.2%, to account for participants anticipating stimuli presentation), where RTs were outside of 2 s.d. of a participant’s mean RT per condition (826 trials, 4.9%), and where a participant’s mean RT in a condition was outside 2 s.d. of the group mean (240 trials, 1.4%). This RT data-cleaning process was necessary to reduce heteroscedasticity and ensure normal residuals from models. This process removed one participant (experiment 1, mean RT = 608 ms), six participant conditions (experiment 1, mean RT = 587 ms) and 1219 trials in total (7.3% of observations) from the RT analysis. The remaining trials had a mean RT of 403 ms. Analysis performed on RT data was done on the inverse of RT to increase normality, and back-transformed when reporting. Choice data was pre-processed by removing trials where no movement past the obstacle was detected, removing 283 trials (1.7% of observations). The extreme obstacle positions were re-coded as −0.9 and 0.9 to indicate the −34.2 and 34.2 mm positions respectively.

Analysis of the choice and RT data was performed using mixed-effect modelling, using the lme4 package in R (v. 1.1–21). Following Barr *et al*. [30], when the maximal random structure did not converge, the optimal random-effects structure was identified using forward model selection, with each mixed-effect model having a random intercept for each participant. The effect of each variable was found using likelihood ratio testing, using the afex package (v. 0.23.0). Post hoc comparisons were performed using the multcomp package (v. 1.4.8), and corrections for multiple comparisons were made using the Bonferroni–Holm method. The MuMIn package (v. 1.43.6) was used to report marginal $R^{2}$ (variance explained by fixed effects), $R_{m}^{2}$ , and conditional $R^{2}$ (variance explained by fixed and random effects), $R_{c}^{2}$ , for the models [31]. The 95% confidence intervals for values are reported in square brackets throughout.

To examine changes in action selection, a mixed-effect logistic regression was performed. The fixed effects were the obstacle’s position (zero-centred), the condition (reference category = random), the experiment (reference category = experiment 1) and all combinations of the interactions between these variables, with a random intercept for each participant. To understand whether hysteresis changed with the experiment, the log odds (LO) of going right around the obstacle at the central obstacle position was compared between conditions, with hysteresis quantified as the increased LO of going right at the central obstacle position in the rightwards condition compared to the leftwards condition. Comparisons were made at this central obstacle position because we would expect the effects of any accumulated bias on choice to be maximally evident when the two choices have the same action cost, and as such any difference in choice at this position should be uniquely related to any accumulated biases. We compared this across experiments. Further, the large sample size in experiment 1 presented an opportunity for more detailed analysis of hysteresis within the random condition. While we expected there would be no global hysteresis in the random condition, a trial-by-trial updating of success probabilities predicts hysteresis at the trial level. To explore this, a mixed-effect logistic regression was performed, with fixed effects of the obstacle’s position (zero-centred), the prime condition (whether the participant went left (left previous) or right (right previous) on the previous trial, with the reference category = left previous), and the interaction between the two, and a random intercept for the participant. Hysteresis here was defined as the difference in LO of going right at the central obstacle position between prime conditions.

To investigate changes in RT, a mixed-effect linear regression was performed. The fixed effects were the trial number in the block (centred on the middle trial in the block), the condition (reference category = random), the experiment (reference category = experiment 1) and all combinations of the interactions between these variables, with a random intercept for the participant and a random slope for condition. To understand how RTs were affected, the estimated marginal mean (EMM) RT at the middle trial in the block was compared between conditions. This should similarly provide the best measure of RT benefits because the middle trial in the block coincides with the central obstacle position for the sequential conditions. The difference in RT between the sequential conditions and random was then compared between experiments. Consistent with choices being biased by the previous trial, we found RTs were shorter when participants repeated their previous action in the random block. To investigate this, a mixed-effect linear regression was performed on the random condition, splitting the data by whether the participant switched or repeated the previous trial’s direction. The model had fixed effects of trial number (centred on the middle trial in the block), switch condition (reference category = repeated), and the interaction of the two, a random intercept for the participant and a random slope of switch condition per participant. The EMM RT at the middle trial was compared between switch conditions.

All statistical analyses and data processing were performed using custom scripts in R. The code and processed data are available at https://osf.io/jcgqk.

### 2.4. A computational model of action selection

A computational model, inspired by POMDPs, was developed where the future selection of an action can be influenced by recent successes and failures through reinforcing successful actions. The model weights the probability of selecting action i (going left or right around the obstacle) by the ‘value’ of each action, $V_{i}$ . We define value as a combination of the expected cost, c , and a bias term accumulated over trials, b , reflecting the increased probability of success from repeating the previous action. These two terms are each normalized by scaling parameters, $s_{c}\;and\;s_{b}$ , respectively, which are used to bring the terms to the same scale so the relative importance of each can be compared. Formally, the ‘value’ of action i is defined as:


(2.1) $$V_{i}=\frac{s_{c}}{c_{i}}+s_{b}\times b_{i}.$$

In this formulation, the expected cost of each action is evaluated as the minimum path length required to avoid the obstacle, and its reciprocal is taken so that higher costs lead to lower action valuations. The action values are converted to the probability of selecting the left action through the softmax function:


(2.2) $$p_{L}=\frac{e^{V_{L}}}{e^{V_{L}}+e^{V_{R}}}.$$

**Figure 2.**
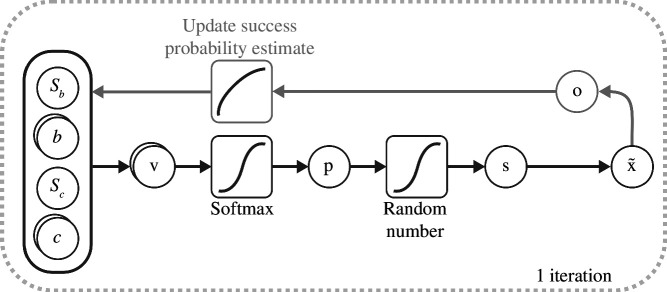
A probabilistic choice model for action selection. In a trial, the ‘value’ for the two actions (going left or right around the obstacle) is calculated from the current costs and biases built up over previous trials. The values are input to a softmax function to generate the probability of selecting the left action. A random number is uniformly sampled and if it is below the probability of the left action then the left is selected, otherwise right is selected. The outcome of executing the associated action is observed and the selection and outcome are used to update the biases for each action according to a reinforcement learning rule.

The left action is selected if a uniformly sampled number between 0 and 1 is below the left action’s selection probability, otherwise the right action is selected. The selected action and outcome of the trial are then used to update the biases associated with each action, according to the following reinforcement learning formula:


(2.3) $$b_{i}=\{\begin{array}{lc} b_{i}+r(1-b_{i}) & if\;i=s,\\ b_{i}+r(0-b_{i}) & \begin{array}{c} if\;i\neq s\\ or\;outcome=fail, \end{array} \end{array}$$

where *r* is the bias rate and *s* is the selected action. It is assumed that participants start with no bias and thus biases are set to 0 on the first trial. While the implementation here focuses on binary decisions about which side of the obstacle to pass, it could instead reinforce a property of the movement performed (like the take-off angle) to accommodate decisions on a continuous scale. A graphical illustration of the model is shown in figure 2.

For simplicity, this model assumed the cost function was the path length of the movement trajectory. Specifically, path length was approximated by fitting the shortest parabola capable of connecting the start point to the target while passing the obstacle on either side. To aid model convergence, path lengths were divided by the minimum possible path length (140 mm). While there has been considerable debate [32] over the quantity used for optimization (including movement cost, endpoint variability, jerk, torque among others), we note that most of these factors co-vary and emphasize that there is a strong tendency for participants to select the shortest possible movement trajectory in unconstrained task settings [33]. Further, the emergence of hysteresis would occur regardless of the exact cost function, so is not critical for the current purpose.

The model was fitted to the choice data for the three experiments using Bayesian estimation via Stan (v. 2.18.2). Each model was fitted using eight chains of 5000 warm-up samples (which were discarded) and 5000 iteration samples, giving 40 000 samples per posterior distribution. Convergence was assessed by visually inspecting chain behaviour and confirming the Gelman–Rubin statistic, $\hat{R}$ , was below 1.1 (maximum 1.01) for all parameters [34,35]. Posterior distributions for each parameter were summarized using the 95% highest density interval (HDI). The empirical priors used for model fitting are shown:


$$\begin{array}{c} s_{c},s_{b}∼N\left(0,25\right)\\ r∼N\left(0.5,0.2\right). \end{array}$$

The priors were designed to be uninformative other than to the scale of the parameter (increasing the width of the priors, i.e. making them less informative, does not affect modelling results). To check the model fit, datasets were simulated based on trial structures sampled from the observed data. For each experiment, 10 000 samples of the posterior distributions were drawn, and each combination was used to simulate a new dataset. Error trials were simulated from extracted error probabilities in the collected data per experiment and obstacle position. On every trial, if a uniformly sampled number between 0 and 1 was below the error rate, the trial was classed as a failed trial and no action was selected. Each new dataset was summarized using a logistic fit per condition. Credible intervals of the model’s predictions were then constructed by taking the minimum and maximum predicted probability of going right around the obstacle at each obstacle position per condition and experiment.

## 3. Results

### 3.1. Choice analysis

**Figure 3.**
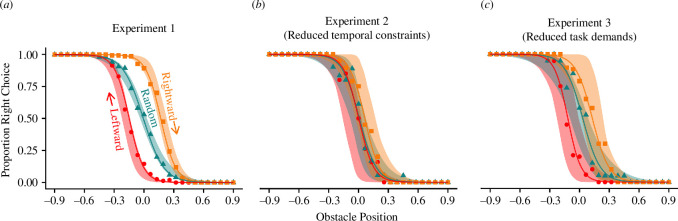
Comparison of experimental and simulated data for choices between experiments. The points and solid lines represent experimental data, with the points indicating the mean proportion of participants who passed the obstacle on the right for each obstacle position, and the solid lines represent the fit of a logistic regression for the experimental condition. The ribbons show the credible range of simulated data from the model, described in §2. The conditions are rightwards (where the obstacle moves from the left of the screen to right between trials), random (where the obstacle moves randomly between trials) and leftwards (where the obstacle moves from the right of the screen to left between trials. (*a*) Experiment 1; (*b*) experiment 2 (reduced temporal constraints); and (*c*) experiment 3 (reduced task demands).

We first examined whether our group-level manipulations resulted in action selection biases. Hysteresis would result in participants going right around the obstacle more often in the rightwards condition (where the obstacle moved from the left of the screen to the right between trials) and going left around the obstacle more often in the leftwards condition (where the obstacle moved from the right of the screen to the left between trials), whereas the random block (where the obstacle moved randomly between trials) should show no overall bias. The biases in decision making are best observed when the obstacle is located centrally, as any difference in choice should be uniquely related to accumulated biases given the cost of either action is identical. We predicted that the degree of this bias would diminish when participants were provided with more planning time (experiment 2) and when action execution was performed under a reduced cognitive task load (experiment 3). The experimental task was successful in revealing hysteresis (figure 3*a*), with the effect diminished in experiment 2 (figure 3*b*) and experiment 3 (figure 3*c*).

We performed a mixed-effect logistic regression to predict the direction participants chose on a given trial. The model (*χ* ^2^_17_ = 18,206.60, *p* < 0.001, $R_{m}^{2}$ = 0.94, $R_{c}^{2}$ = 0.95) revealed a significant main effect of obstacle position (*χ* ^2^_1_ = 4627.52, *p* < 0.001) and condition (*χ* ^2^_2_ = 1330.53, *p* < 0.001), but no significant effect of experiment (*χ* ^2^_2_ = 2.61, *p* = 0.272). There were significant interactions between position and condition (*χ* ^2^_2_ = 54.01, *p* < 0.001), and condition and experiment (*χ* ^2^_4_ = 94.53, *p* < 0.001), but no significant interaction between position and experiment (*χ* ^2^_2_ = 4.60, *p* = 0.100), or between position, condition and experiment (*χ* ^2^_4_ = 4.36, *p* = 0.359). The interaction between condition and experiment suggested that the choice at the central obstacle position changed between both conditions and experiment.

To further explore this, Bonferroni–Holm corrected comparisons were performed to see how the LO of passing the obstacle on the right changed with condition and experiment at the central obstacle position. In experiment 1, participants were significantly more likely to go right in rightwards compared to random (LO = 2.52 [2.18–2.86], *p* < 0.001), and significantly less likely to go right in leftwards compared to random (LO = −2.18 [-2.51 to -1.85], *p* < 0.001), indicating a persistence in use of the previous action in the sequential conditions. Participants were more likely to go right in rightwards compared to leftwards (LO = 4.70 [4.28–5.12], *p* < 0.001); this comparison gives the difference in choice between the two sequential conditions and is the measure of hysteresis used throughout.

In experiment 2, where participants were forced to wait at the start point for 1.5 s while the obstacle was shown, they were significantly more likely to go right in rightwards compared to random (LO = 0.82 [0.05–1.59], *p* = 0.026), but not in leftwards compared to random (LO = −0.07 [−0.80 to 0.65], *p* = 0.811). Furthermore, participants were more likely to go right in the rightwards trials compared to leftwards (LO = 0.89 [0.11–1.68], *p* = 0.023), indicating hysteresis was present, but numerically smaller than in experiment 1.

In experiment 3, where participants were presented with a reduced cognitive task load, they were significantly more likely to go right in rightwards compared to random (LO = 1.24 [0.44–2.03], *p* < 0.001), and significantly less likely to go right in leftwards compared to random (LO = −2.30 [−3.21 to −1.39], *p* < 0.001). Participants were also more likely to go right in rightwards compared to leftwards (LO = 3.54 [2.54–4.54], *p* < 0.001), again indicating hysteresis was present but at a smaller magnitude than in experiment 1.

To formally test whether the magnitude of hysteresis changed between the experiments, the increased LO of going right in the rightwards condition compared to the leftwards condition were compared across experiments. Participants in experiment 2 showed significantly less hysteresis than participants in experiment 1 (LO = −3.81 [−4.70 to −2.93], *p* < 0.001), and participants in experiment 3 also showed significantly less hysteresis than participants in experiment 1 (LO = −1.16 [−0.09 to −2.24], *p* = 0.012). These results indicate that the experiment interventions designed to decrease temporal constraints and decrease cognitive task load both reduced the magnitude of hysteresis in experiments 2 and 3, respectively (relative to experiment 1), with a larger attenuation for planning time constraints (experiment 2).

Simulations from the action selection model were fitted with the experimental data. The 95% HDI of the posterior distributions for each parameter are summarized in table 1. To understand whether the bias changed between experiments, the posterior distribution for the bias scaler parameters in experiments 2 and 3 were subtracted from that of experiment 1. This showed that the bias scaler estimate in experiment 2 was lower than in experiment 1 (mean difference = −1.60, 95% HDI = [−1.18 to –2.02]), and the estimate in experiment 3 was also lower than in experiment 1 (mean difference = −0.82, 95% HDI = [−0.38 to –1.25]), indicating hysteresis was attenuated by a reduced contribution of biases, built up through previous successes and failures, towards action selection under reduced temporal constraints and cognitive load.

**Table 1.** Mean and 95% HDI estimates of the decision-making model parameters.

	mean [95% HDI]		
experiment	cost scaler	bias scaler	bias rate
experiment 1	39.26 [37.36–41.24]	2.69 [2.53–2.86]	0.32 [0.28–0.36]
experiment 2 (reduced temporal constraints)	41.96 [36.59–47.84]	1.09 [0.71–1.48]	0.43 [0.27–0.62]
experiment 3 (reduced task demands)	37.63 [32.94–42.82]	1.87 [1.48–2.29]	0.35 [0.21–0.50]

These posterior distributions were then used to simulate new data so that predictions from the model could be compared to the experimental data, as described in §2, and are shown as the shaded ribbons in figure 3. Note that the observed selection probabilities lie within the range of the model’s predictions, with distinct separations between the two sequential conditions for experiments 1 and 3 around the central obstacle positions, but for experiment 2, the two sequential conditions share considerable overlap, indicating that the model produces hysteresis and shows predictions consistent with the observed data.

**Figure 4.**
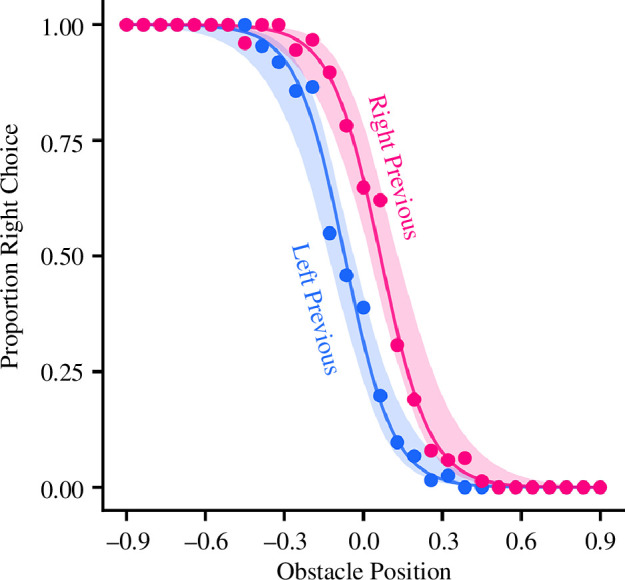
Empirical and simulated data for choices in experiment 1 random condition. The points indicate the mean proportion of participants who passed the obstacle on the left for each obstacle position, and the solid lines represent the fit of a logistic regression for the experimental condition. The ribbons show the credible range of simulated data from the model, described in §2. The conditions are left previous (where the participant passed the obstacle on the left on the previous trial) and right previous (where participants passed the obstacle on the right on the previous trial).

The results thus far indicate participants are biased towards repeating previously used action structures when the obstacle moves between trials in a sequential manner. While most hysteresis studies employ similar sequential trial designs, some have also found hysteresis when stimuli are varied randomly across trials [14,21]. Thus, we explored whether the participant’s selection on a current trial was biased by the direction they passed the obstacle on the previous trial in the random block. Analysis of the data for repeated and switched trials from the random block in experiment 1 provided support for this idea (figure 4).

A mixed-effect logistic regression was performed on data from the random block from experiment 1 to predict the direction participants went in the current trial. The model (*χ* ^2^_3_ = 4406.80, *p* < 0.001, $R_{m}^{2}$ = 0.91, $R_{c}^{2}$ = 0.93) showed a significant main effect of position (*χ* ^2^_1_ = 2215.09, *p* < 0.001) and prime condition (*χ* ^2^_1_ = 96.62, *p* < 0.001), but there was no significant interaction between position and prime condition (*χ* ^2^_1_ = 0.16, *p* = 0.688). To explore the main effect of the prime condition, the LO of passing the obstacle on the right were compared between prime conditions. Participants were significantly more likely to go right in the right previous condition compared to the left previous condition (LO = 1.51 [1.19–1.83], *p* < 0.001). The increased LO of going right were smaller for the two prime conditions when compared to the two sequential conditions from the analysis reported above—suggesting biases accumulate over longer action sequences than just the previous trial. Predictions of the model, based on the parameter posterior distributions from experiment 1, are shown as ribbons in figure 4. Consistent with the observed experiment data, the model shows a distinct separation between the two prime conditions, with right previous cases being more likely to go right at the central obstacle positions.

### 3.2. Reaction times analysis

**Figure 5.**
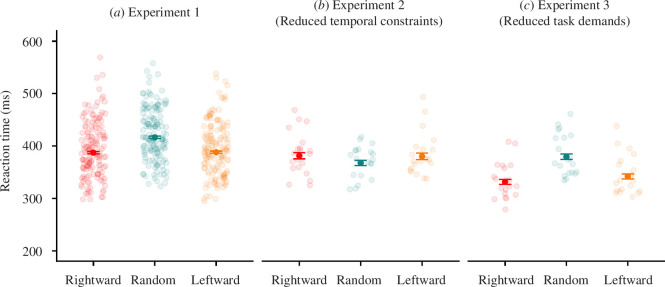
Comparison of RTs for each condition between experiments. The open circles show the mean RTs for each participant. The solid circles show the mean RT for each combination of conditions and experiment across all participants, and the error bars show the 95% confidence intervals around the estimate of the mean. The conditions are rightwards (where the obstacle moves from the left of the screen to the right between trials), random (where the obstacle moves randomly between trials), and leftwards (where the obstacle moves from the right of the screen to the left between trials). (*a*) Experiment 1; (*b*) experiment 2 (reduced temporal constraints); and (*c*) experiment 3 (reduced task demands).

Participants in experiments 1 and 3 showed a reduction in RT in the sequential conditions compared to the random condition, but RTs in experiment 2 seem similar across conditions, with a slight elevation for the sequential conditions (figure 5). To understand how RTs were affected by biases, a linear mixed-effect model was conducted to predict RTs on a given trial. The model (*χ* ^2^_22_ = 2337.69, *p* < 0.001, $R_{m}^{2}$ = 0.10, $R_{c}^{2}$ = 0.49) showed a significant main effect of trial (*χ* ^2^_1_ = 7.53, *p* = 0.006), condition (*χ* ^2^_2_ = 104.22, *p* < 0.001) and experiment (*χ* ^2^_2_ = 30.30, *p* < 0.001). There were significant interactions of trial and condition (*χ* ^2^_2_ = 28.45, *p* < 0.001), and condition and experiment (*χ* ^2^_4_ = 50.58, *p* < 0.001), but no significant interaction between trial and experiment (*χ* ^2^_2_ = 2.47, *p* = 0.291). There was a significant interaction between trial, condition and experiment (*χ* ^2^_4_ = 19.72, *p* < 0.001).

To explore the interaction of condition and experiment (i.e. the difference in RT between conditions changed with experiment), Bonferroni–Holm corrected comparisons were performed to see how RTs changed with condition and experiment at the middle trial in the block. The middle trial in the sequential conditions coincides with the obstacle being located centrally, so we would also expect the effect on RTs to be best observed there. In experiment 1, RTs in the random condition (EMM = 417 ms [410–425]) were significantly higher than in the leftwards (EMM = 389 ms [381–397], *p* < 0.001) and rightwards conditions (EMM = 387 ms [380–396], *p* < 0.001), with no significant difference between the leftwards and rightwards conditions (*p* = 0.659). The reduced RTs (approx. 30 ms) in the sequential conditions suggest an RT saving from choice perseveration. In experiment 2, there were no significant differences between the random (EMM = 368 ms [352–385]) and leftwards condition (EMM = 380 ms [361–402], *p* = 0.151), the random and rightwards condition (EMM = 382 ms [361–404], *p* = 0.151) or between the leftwards and rightwards condition (*p* = 0.877), indicating the attenuation of hysteresis accompanied a lack of RT benefits. In experiment 3, the random condition (EMM = 380 ms [363–398]) was significantly slower than the leftwards (EMM = 342 ms [326–359, *p* < 0.001) and rightwards conditions (EMM = 331 ms [316–348], *p* < 0.001), but there was no significant difference between the leftwards and rightwards conditions (*p* = 0.093). Participants were 38–49 ms quicker in the sequential conditions than in the random condition, showing savings that were marginally larger than those observed in experiment 1.

To investigate whether the magnitude of RT reduction changed between experiments, we compared the difference in RT of the random condition to the sequential conditions between experiments. The difference in RT between the random and leftwards conditions was significantly lower in experiment 2 compared to experiment 1 (*p* < 0.001), but significantly higher in experiment 3 compared to experiment 1 (*p* < 0.001). The difference in RT between the random and rightward conditions was significantly lower in experiment 2 compared to experiment 1 (*p* < 0.001), but significantly higher in experiment 3 compared to experiment 1 (*p* = 0.016).

**Figure 6.**
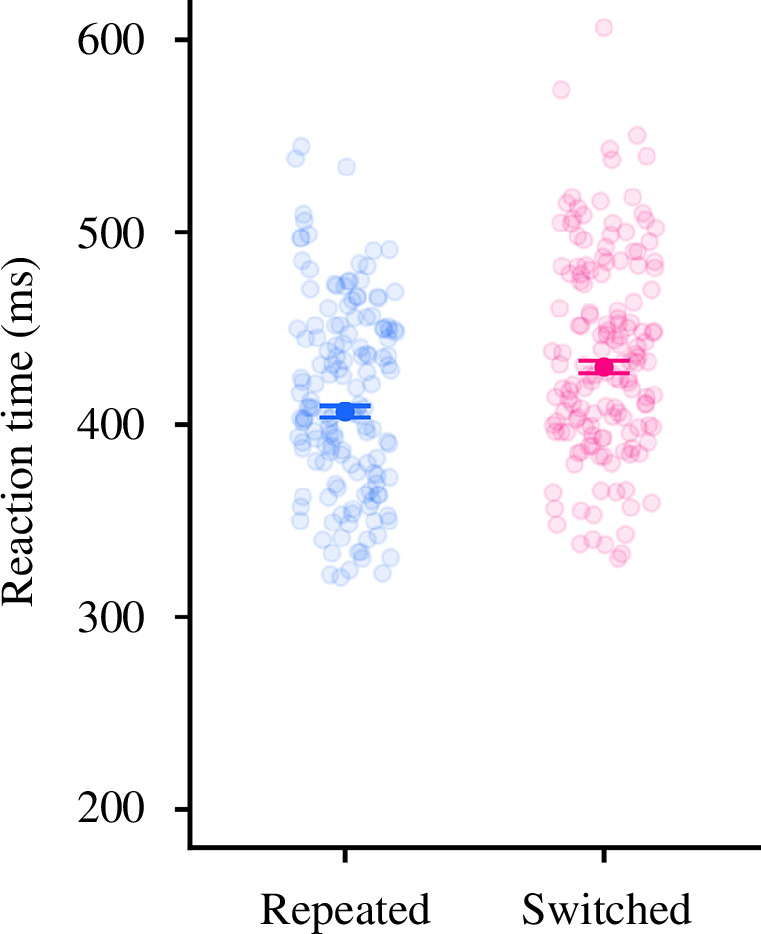
Comparison of RTs inside experiment 1’s random condition. The open circles show the mean RTs for each participant. The solid circles show the mean RT for each switch condition across all participants, and the error bars show the 95% confidence intervals around the estimate of the mean. The conditions are ‘repeated’ (where participants made the same choice on the current trial as on the last trial) and ‘switched’ (where participants switched choice from the previous trial).

In the random block of experiment 1 participants showed a reduction in RT for repeated choices compared to switched choices (figure 6). To understand how RTs changed with switch condition on a given trial, a linear mixed-effect model was performed. The model (χ^2^_5_ = 197.71, *p* < 0.001, $R_{m}^{2}$ = 0.03, $R_{c}^{2}$ = 0.48) showed a significant main effect of trial (*χ* ^2^_1_ = 14.23, *p* < 0.001) and switch condition (*χ* ^2^_1_ = 97.79, *p* < 0.001), but no interaction between trial and switch condition (*χ* ^2^_1_ = 0.19, *p* = 0.663). A comparison was performed to see how RTs changed with switch condition at the middle trial in the block. The repeated condition (EMM = 407 ms [400–415]) was significantly faster than the switched condition (EMM = 429 ms [421–438], *p* < 0.001), indicating participants had an RT benefit from repeating a single previous choice. The difference between the switched and repeated conditions was lower than the difference between the random and the sequential conditions from the earlier analysis, indicating RT savings may accumulate in a similar way to the choice biases.

## 4. Discussion

Our goal was to examine the bias shown by skilled adults towards selecting a previously selected action structure when alternative options that would be selected in de novo conditions were available. To this end, we created a simple obstacle avoidance aiming task, where participants had to move around an obstacle to reach a target. We found participants exhibited hysteresis effects when the obstacle moved systematically in one direction between trials, with participants more likely to continue moving around the obstacle on the side they did previously. When the obstacle moved randomly between trials there was no global hysteresis effect, but a trial-by-trial analysis showed choices were still biased by the previous trial. Further, RTs were lower in the sequential conditions, when compared to the random condition, indicating an RT reduction co-occurred with hysteresis effects. We were also interested in exploring the impact on the hysteresis of changing the temporal constraints of the task, motivated by findings that decision making is underpinned by a process of evidence accumulation. We used two manipulations to alter the temporal constraints of the task. In experiment 2, we directly manipulated the temporal constraints by preventing action until a 1500 ms time window had elapsed. In experiment 3, we indirectly altered the constraints by decreasing the cognitive demands of the task (reasoning that less time spent identifying the task goal would provide more time for evaluating the respective costs of the available actions). The results showed that the hysteresis effect was practically eliminated in experiment 2 and attenuated in experiment 3.

Our investigation of hysteresis was motivated by our hypothesis that hysteresis is the naturally emergent property of a learning system that is operating in an uncertain world. In order to test our hypothesis we created a simple POMDP-inspired computational model that incorporated dynamic probabilistic estimate updating. We used this model to simulate behavioural responses for the experimental tasks and found that it was able to capture the empirical data. This finding suggests hysteresis can be explained as the byproduct of a relatively simple principle—that humans weight current actions by recent successes and failures—rather than assuming its purpose is to increase computational efficiency. The results emphasize the dynamic nature of human sensorimotor decision making. The challenge for the human nervous system is to maintain optimal action selection in a noisy and uncertain world which can be accomplished through an ongoing evaluation of the accuracy of its internal representation of the external world, and frequent updating of its probability estimates. The current findings suggest that this updating occurs on a trial-by-trial basis, with biases towards recently used actions accumulating over trials, consistent with findings on choice hysteresis [4]. This paints a picture of a system that is continually adapting and ensuring that its actions are precisely tailored to the external environment.

Our experiments have focussed on the sensorimotor system, but the general phenomenon of hysteresis can be observed in other aspects of human behaviour. For example, hysteresis effects have been observed in perceptual decision-making tasks where choices are biased by previous decisions [36–38], and the magnitude of the perceptual bias tends to depend on whether the previous decision was rewarded or not [36,39]. These hysteresis effects (typically described as choice-history biases) have been successfully implemented in evidence accumulation models of decision making, whereby recent choices influence the initial amount or rate of evidence accumulation towards an option. We argue that the existence of hysteresis within the perceptual system can be explained through the same mechanisms that we used to account for hysteresis in sensorimotor decision making. In line with the existence of evidence accumulation processes, we found that the hysteresis effect was attenuated when the temporal constraints of the task were eased, with the greatest attenuation occurring when participants were forced to wait before moving. This emphasizes the tendency within the system to select the first action to cross a pre-specified threshold when under pressure to do so, favouring recently successful actions, rather than waiting until the full costs of all options have been exhaustively evaluated.

It is also possible that similar mechanisms can account for reports of hysteresis in higher-order cognition (often described as ‘perseverance’). For example, perseverance has been observed in reinforcement learning tasks, where it is modelled in a similar manner to our bias here [40–42], or interpreted as the interplay between goal-directed and habitual behaviour [43]. There is a growing consensus that the sensorimotor system provides the phylogenetic and ontogenetic foundations of higher-order cognition [44,45], and the postulated links between the sensorimotor and cognitive system might suggest a close relationship between sensorimotor hysteresis and perseveration type behaviours. This may prove a fruitful line of investigation for future studies.

The presented model provides a parsimonious account of how hysteresis can emerge from a simple principle of reinforcement. There are several areas where future research could investigate and extend this model to account for a wider range of behavioural effects. To bring the model more in line with the evidence accumulation framework, the decision rule used could be changed to an evidence accumulation process (e.g. the drift-diffusion model [46]), which would allow the joint modelling of the effect of biases on choice and RT data. Furthermore, whether an action is (i) not selected or (ii) selected but leads to an error is currently handled identically, but it seems unlikely that this would be the case. Studies on perceptual decision making have found complex mechanisms by which errors influence behaviour, for example, one study found errors temporarily gate accumulated biases before showing a rebound to existing levels following a correct response [39]. Future work could be tailored to investigate the effect of errors on sensorimotor hysteresis and how this may be best modelled.

Previous accounts of hysteresis have assumed that the sensorimotor system has a ‘hysteresis’ function whose purpose is to create an advantage when planning a new movement. It is argued that modifying a previously used plan would be more cognitively efficient than planning a new one from scratch, so hysteresis exists to improve planning efficiency [11,18–20], as indexed by reduced RTs when performing the same action as previously [21]. Based on previous reports, we fully expected to find reduced RTs when the hysteresis effect was present. Moreover, we anticipated the presence of reduced RTs on the theoretical basis that evidence accumulation processes will cause actions to be selected more rapidly when there is a bias towards one action versus another. In line with these expectations, we observed a decrease in the average RT on the sequential trials in experiment 1 relative to the random trials. The quantitative relationship between choice and RT hysteresis could not be assessed within the current work as the short experiment durations meant hysteresis was not well characterized for each individual, instead relying on group-level comparisons (though we note this correlation has been found previously for hand choice [21]). In experiment 2, participants were given a substantial time to select the goal-directed action and the RTs were similar across conditions. The reduced task demands in experiment 3 were accompanied by a general reduction in RT, while still showing benefits in the sequential conditions. We observed reduced choice hysteresis in experiment 3 relative to experiment 1, but increased RT benefits. It may be possible that task complexity interacts with evidence accumulation processes in a non-trivial way (e.g. influences several parameters such that the distributions of process outcomes are further away on average but overlap more) or influences other processes (e.g. turning choice into action). Future work designed to quantitatively relate choice and RT hysteresis (e.g. by assessing the previous trial effect in an extensive random condition) would afford the opportunity to investigate the association further and directly test an evidence accumulation model, including understanding any departures from it.

The work presented within this manuscript addresses issues from the field of ‘human-like-computing’ where researchers attempt to bridge the gap between models of human decision making and the models used in artificial intelligence and robot motion control. Stochastic models of actions, observations, costs and rewards are the main tools used in modelling and planning robot motion, including tasks that involve reaching behind obstacles [2]. An improved understanding of human decision making can inform the development of such robot motion models. The identification of the hysteresis bias allows roboticists and computer scientists to decide whether their agents are operating within environments that are sufficiently constrained so that control schemes can seek to ameliorate hysteresis. Alternatively, hysteresis may suggest mechanisms through which a robotic agent can show human-like flexibility and adaptability in complex dynamic environments. Moreover, the identification of hysteresis as an emergent property can help improve the legibility and predictability available within human–robot interactions. It follows that investigations into human biases (such as hysteresis), and their formal description through mathematical models, can be useful in robot motion and control. Thus, the approach adopted within this manuscript provides an interesting avenue for future investigations by roboticists, psychologists and computer scientists.

## Data Availability

The code and processed data are available at [47].
